# Hamamelis Virginica

**Published:** 1867-07

**Authors:** W. W. Durham


### *Hamamelis Virginica.*

The following communication, from Dr. W. W. Durham, attributes to this plant properties not generally known to the profession. While the readers of the JOURNAL are testing the reputed virtues of black-haw it may be that the witch-hazel will be found valuable in this way:

"I will in the first place, in the articles I propose to write for your journal, bring to your notice the witch hazel or *hamamelis virginica.* It possesses properties in common with the black haw, or *viburnum prunifolium*, that is the property of preventing "abortion or miscariage. At one period of my practice the negroes used the cotton root so frequently to produce abortion that my supply of black-haw become exhausted, and having heard of this power of the hazel to effect the purpose for which I used the haw. I resorted to it (the hazel) with perfect succes—having only used it for the purpose of preventing abortion, from the effects of the cotton root: I cannot speak of it in other cases.

Its valuable action being generally well known, otherwise than in the case to which I have alluded, I will not speak of it.

For the purpose of which I have spoken, steep one ounce of the leaves in one pint of water, and drink freely."

W. W. DURHAM.

